# Evaluation of the Patient Innovation Partner Role: Perceived Benefits, Structures, Supports, and Recommendations for Lived Experience Engagement in Healthcare Innovation Teams

**DOI:** 10.1111/hex.70194

**Published:** 2025-03-27

**Authors:** Shoshana H. Bardach, Amanda Perry, Terry Sturke, Randy Stevens, Milan F. Satcher

**Affiliations:** ^1^ The Dartmouth Institute for Health Policy & Clinical Practice, Geisel School of Medicine at Dartmouth Hanover New Hampshire USA; ^2^ Family Heart Foundation Fernandina Beach Florida USA; ^3^ Hope for New Hampshire Recovery Manchester New Hampshire USA; ^4^ Community & Family Medicine, Dartmouth Health Lebanon New Hampshire USA

**Keywords:** healthcare innovation, human‐centered design, lived experience, patient partner, quality improvement

## Abstract

**Background:**

Patient engagement plays a valuable role in health research and quality improvement. While prior research highlights some principles and key considerations for patient involvement in these efforts, there is a limited understanding of how best to structure and support this engagement, especially from the patient perspective and for healthcare innovation projects.

**Methods:**

Transcripts and notes from semi‐structured debrief interviews with patient partners (*n* = 14) and team leads (*n* = 22) of 14 healthcare innovation projects conducted between 2020 and 2023 were analyzed thematically to identify perceived benefits, structures and supports that shape lived‐experience engagement, and recommendations for future patients' participation in healthcare innovation teams.

**Results:**

Lived‐experience engagement was perceived as highly valuable to project teams and rewarding to the patients themselves. Approaches for structuring and supporting the patient role shaped engagement, highlighting several strategies (e.g., providing patient partners with opportunities to reflect and prepare, having smaller check‐ins, truly getting to know the patient, and offering opportunities for in‐person connection) to enhance the experience and ameliorate challenges. Patients also emphasized the importance of sharing their perspectives to fully realize the benefits of their engagement.

**Conclusion:**

These findings highlight the importance of recognizing the bidirectional benefits of patient engagement within project teams. Taking opportunities to check in with patients throughout the project period, both formally and informally, regarding their preferences for involvement and experiences on the team would enable real‐time feedback and adjustments to optimize patient partner engagement.

**Patient or Public Contribution:**

Since its inception, the Susan and Richard Levy Healthcare Delivery Incubator has incorporated patient and public involvement into the design and operations of its healthcare innovation projects. While the conceptualization of this analysis did not engage patients or the public, patients and individuals with lived experience provided the data. Further, three patient partners were engaged in the review of the findings, two of whom also actively contributed to the preparation of the manuscript by reviewing drafts, adding content, and making revisions.

## 1 Background

There is increasingly a recognition of the value of incorporating the patient perspective into health research and quality improvement initiatives, strengthening the relationship between resources, research and patient needs, with some funding agencies even requiring such engagement [1, 2, 3]. Experience‐based codesign is an approach that highlights the importance of involving patients, family members, and healthcare providers throughout the project process [4]. Prior research suggests principles (e.g., respect and trust‐building) and considerations (e.g., financial compensation and ongoing dialogue) that can optimize the codesign experience and maximize the benefit of patient input [5]. A systematic review of patient partner engagement in research studies found this engagement was typically considered a positive experience but that challenges also emerged [6]. Challenges included when patient partners felt ill‐prepared for engagement or when power differentials impeded participation [6]. Another review focused on the impact of patient partnership on research outcomes similarly identified power imbalances as a threat to the experience and impact of patient engagement, and highlighted the importance of valuing the patient partner's engagement and being mindful of jargon [7]. While there is some discussion of how to compensate patients [8], there is limited attention to how best structure and support overall patient engagement and opportunities to better understand the experiences of those with lived experience as patients working in conjunction with healthcare professionals and researchers.

### 1.1 The Incubator—Overview

The Susan and Richard Levy Healthcare Delivery Incubator (The Incubator) is a partnership between Dartmouth College and Dartmouth Health that provides 1 year of funding, staff support and educational guidance on the principles of design‐thinking to facilitate the rapid design and implementation of healthcare delivery innovations for various populations [9]. Innovation in this context is conceptualized as creating new ways of delivering care rather than simply making incremental improvements to existing processes [10]. The Incubator was founded in 2019, with an initial focus on populations experiencing serious illness, but subsequently expanded its scope to improving care for patients, families, and care teams without the serious illness requirement. The Incubator was designed with several goals: to improve the patient experience, prioritize a patient‐focused approach, advance quality improvement of services delivered, reduce spending, and advance scholarly work.

### 1.2 The Incubator—The Patient Innovation Partner Role

Incubator teams are selected by a competitive, community participatory, peer‐review process and are comprised of healthcare providers, healthcare staff, college faculty, community representatives, and individuals with lived experience with the target condition. The individuals with lived experience are titled ‘patient innovation partners‘ (PIPs) to reflect their active role in the design process; teams are required to include at least one PIP. We use the term PIP throughout this manuscript to include all individuals with lived experience who served in this role, acknowledging that diverse perspectives beyond experiences as a patient were represented, respected, and valued.

Through the incubator process, PIPs engage in the weekly team meetings and help keep the lived experience front and center throughout the codesign process. PIPs are frequently identified through the team lead's clinical work and are provided with a quarterly stipend to compensate them for their time. All team members, including PIPs, engage in a retreat at the beginning of the project year that includes fundamentals of design‐thinking, incorporates team‐building activities, introduces healthcare innovation concepts, and previews the work and year to come; these areas are all then reinforced by Incubator staff throughout the project year.

### 1.3 Purpose

The Incubator, now in its 5th year, has consistently engaged PIPs, but has not yet formally evaluated how to optimize this role. The PIP role is somewhat distinct from patient or community member engagement in research projects or quality improvement initiatives; the innovative nature of the Incubator's work—where the goals and strategies of projects evolve as needs and opportunities are better understood—introduces a level of unpredictability typically not present in more prescribed projects. This ambiguity may make it harder to clearly delineate roles, but also creates space for creative thinking and novel perspectives to meaningfully shape the way projects develop. The purpose of this study is to evaluate the perceived benefits of including PIPs on healthcare innovation teams, the structures and supports that shape PIP engagement, and recommendations for future PIPs' participation in such teams.

## 2 Methods

### 2.1 Approach

Following the completion of each project's year‐long funding period, the Incubator staff sent email invitations to all team leads and PIPs to participate in a semi‐structured interview via Zoom to debrief their experience. Interviews were conducted by Incubator staff (SHB and AP), both of whom had training and experience with qualitative methods and had worked with all interviewees over the project year. Interviews were framed as an opportunity to provide feedback to guide the Incubator's future work. Accordingly, both positive and negative feedback was explicitly requested. Interviewers shared their desire to learn and improve from the feedback received. At the conclusion of the interviews, interviewees were told they could reach back out if they had additional feedback they would like to share, but no one chose to participate in a follow‐up interview.

Interviews were recorded through the Zoom platform and interviewers also took notes to capture responses and insights. Interviews were designed to be brief and low burden. PIPs were asked about how they became engaged in the team, what roles they played in the project, their experiences and comfort contributing to the work, what they most enjoyed, if they felt they provided value, and suggestions for what would have improved the experience and anything they wish they had known before participating. PIP interviews were typically around 15 min. Team lead interviews tended to be longer, closer to 40 min, as they covered more topics. Team lead questions pertaining to the PIP role included questions about their overall experience working with the PIP(s), how engaged they felt the PIP was, and how the PIP provided value to the project. For this study, we reviewed the transcripts and notes from interviews with the first four cohorts of Incubator teams, including team leads/co‐leads and PIPs. The Dartmouth Health Institutional Review Board reviewed the project procedures and determined this work to be nonhuman subjects research and part of a quality improvement initiative.

### 2.2 Analysis

A thematic qualitative analysis approach was used to analyze the data, starting deductively with pre‐existing broader categories based on the interview questions, and inductively identifying subthemes within these categories [11]. Three of the authors, including Incubator staff members (SHB and AP), and a former team lead (MFS) reviewed the interview notes and transcripts in entirety, noting benefits of PIP engagement, challenges and opportunities for improvement, and advice for future PIPs mentioned in each interview. Segments of each interview that addressed these areas were collated into a separate document and sorted, using a constant comparative approach to identify similarities and differences and enhance understanding of PIP engagement. Initial extraction and sorting was conducted by the first author (SHB); two additional authors (AP and MFS) reviewed the full transcripts to ensure data segments were fully extracted and appropriately categorized, and added any overlooked segments and made adjustments to sorting [12, 13]. Subthemes were reviewed and finalized among these three authors (SHB, AP and MFS) to ensure consensus. Three individuals who had served as team PIPs, two of whom are co‐authors (TS and RS), reviewed the thematic findings to ensure they resonated and added clarification and elaboration, as a form of member checking to enhance credibility, rigour and partnership (see Table 1 for an overview of the analytic process) [14].

**Table 1:** Overview of analytic process.

Stages	Activities
Step 1. Summarize the interviews	Take notes following each interview, highlighting key quotes and insights Review Zoom transcript of each interview, make corrections as needed based on audio file and notes Create a summary compiled document of all transcripts
Step 2. Code the interviews	Manually code text segments using Microsoft Word that reflect benefits, challenges, and opportunities relating to PIP engagement in healthcare innovation teams' work Using the deductive categories as an initial scaffold, segments were extracted from transcripts and sorted into a new document, moving quotations around as needed to reflect growing understanding, comparing and contrasting with existing quotations to group them conceptually and highlight commonalities and new insights
Step 3. Synthesize	Summarise and describe findings, discussing when multiple meanings or interpretations are possible
Step 4. Review and member check	Review summary compiled document of all transcripts to identify any omissions in content Review, clarify, and establish consensus regarding the emerged subthemes, moving segments as needed and adding explanation/meaning as appropriate

## 3 Results

### 3.1 Participant Description

Interviews were conducted with a total of 22 team leads/co‐leads, and 14 PIPs across the 14 projects conducted in the first four cohorts of teams (2020–2023). This represented all team leads/co‐leads and the majority (14/16) of PIPs engaged in these teams. The two PIPs who did not participate in the interviews both indicated an initial willingness, but subsequently did not respond to requests for scheduling. The 14 PIPs interviewed were predominantly female (78.6%, 11/14) and Non‐Hispanic White (92.9%, 13/14). PIPs brought a range of life experiences to the project teams, including: experience living with and receiving healthcare for conditions such as heart failure, stroke, serious infections resulting from injection drug use, lung cancer, post‐acute COVID‐19, inflammatory bowel disease, and Crohn's disease; experiences as caregivers for infants requiring neonatal intensive care and care partners for older adults requiring surgical care; and experiencing unique life challenges and stressors such as re‐entering the community following incarceration and working in a Medical Intensive Care Unit. The team leads/co‐leads were more evenly split on gender, with 59.1% female (13/22) and were similarly predominantly Non‐Hispanic White (90.9%, 20/22). All team leads/co‐leads had advanced graduate degrees, with all but two serving in predominantly clinical (medicine or psychology) roles; of the remaining two one had a clinical background but was working predominantly in a quality role and the other was a medical anthropologist.

### 3.2 Findings

The interviews with PIPs and team leads highlighted numerous benefits of PIP engagement, structures and supports that shape PIP engagement, and advice for future PIPs. Benefits of PIP engagement included: benefiting the project work by bringing an enhanced understanding of the lived‐experience perspective and providing motivation/highlighting the importance of the work, PIP experiences of feeling valued and making a difference, leaving many with a desire to engage further. Structures and supports that shape PIP engagement included providing clarity on when and how to contribute to create comfort, recognizing the impact of virtual meetings, and the bi‐directional need to adjust to different norms and schedules. Recommendations to future PIPs centered around the importance of recognizing value and being willing to speak up and contribute, while also acknowledging the work can be emotionally demanding. These are each discussed in detail below.

#### 3.2.1 Theme 1: Benefits of PIP Engagement

*1a. Benefiting Innovation Project Work*

PIPs and team leads consistently indicated that the PIP(s) played a valuable role in the project team. One team lead shared her perspective, noting, ‘[the PIP] has been invaluable to the project…I don't think we could have done it without her.’ Numerous benefits for the team and the project work were discussed, including helping the team to understand how individuals with the target condition may interpret or experience things, how different wording choices might be interpreted, generating ideas, and considering the feasibility and acceptability of various intervention ideas. PIPs' perspectives also helped teams understand how social determinants and factors outside of healthcare shape individuals' ability to navigate healthcare systems, advocate for their own needs, and influence decisions about disease management and self‐care. In addition, team leads shared how PIPs helped provide a source of motivation—both for the team itself and for external groups that the team engaged with, for example, primary care providers. One team lead shared, ‘Having him [the PIP] speak at meetings was a highlight.’ Another team lead spoke about how the PIP fuelled her motivation, ‘even when the work seemed more difficult, I was not going to stop because it was so important to her.’ The ability to share a personal story helped to emphasize the real‐world relevance and human impact of the team's work and reinforced the team's desire to make a positive difference for the target population.

*1b. Feeling Valued*

PIPs also shared that they found the experience to be enriching and rewarding, beyond their expectations, sometimes challenging them to think in new ways. One PIP shared, ‘we went further than I had dreamed we would.’ Another PIP shared that the team was composed of ‘very smart, motivated, interesting people. Everybody worked hard to a common goal…I was pretty energized by the whole project’. Another PIP expanded on appreciating the experience of being part of a motivated, passionate, diverse team and appreciating having their own voice heard:

What I enjoyed most about working on this was actually just witnessing the meetings of the minds. Being able to see so many individuals across so many different diverse areas or networks, or parts of the industry coming together to work on a common goal, or a solution that benefits humans as a whole. That for me…was so refreshing. I enjoy coming and seeing so many different diverse individuals all actively work together on bringing a solution to the community…What I enjoyed was the fact that [other team PIPs] and other directly impacted voices were centered in the thinking of how to move forward. It was valued.

PIPs frequently expressed this sentiment of feeling valued. One PIP stated, ‘I always felt that I was a full member of the team, and I was respected, as we each were, for our unique contributions.’

*1c. Making a Difference*

PIPs often shared that they appreciate really creating change, for example, it was ‘good to be on a project that actually implemented [ideas]. Not just making mission statements and stuff’. Another PIP similarly expressed appreciation for making real‐world impact, noting, ‘I liked to see the process as it unfolded. [I] was so excited when [we] had [our] first patient…Knowing I was part of something, a whole new program that is going to help people. That was just great.’ This sentiment of valuing being a part of care innovation that will improve lives was expressed by PIPs across interviews. One PIP expressed, ‘to [give] some sliver of help in that is really cool.’ Another indicated, ‘paying it forward, being able to use my experience and having a positive influence was important,’ and another shared, ‘[I] liked affecting change in a positive way.’ In general, PIPs indicated that recognizing that their thoughts influenced design, ‘felt good’ and enabled them to feel valued.

*1d. Fueling a Desire to Engage*

Given the positives of their experiences, PIPs were often left with a desire to do even more—either on the current project or in future work. One PIP shared, ‘I wish we could do more. Wish we could continue in some capacity to help them, or help in any way, because we are so thankful.’ Another PIP shared, ‘I want to do it again. I really do. It was an important part of my year.’ Many PIPs valued the opportunity to see how much clinical teams truly care about patients with their lived condition; this motivated some PIPs to act as informal ambassadors to raise awareness of the innovations among their patient communities; as one PIP stated, ‘just reassuring…to be able to see the behind the scenes stuff, and to know like it's not like an empty abyss… I've been really quick to say that to people because I have friends… they didn't know that Dartmouth itself was doing stuff… I was always like, “No, they are. You just can't see it yet”.’

#### 3.2.2 Theme 2: Structures and Supports That Shape PIP Engagement

*2a. Clarify Contribution Expectations (What, When, How, Who, Why) to Create Comfort for Engagement*

The way the PIP role is established and structured may influence the extent to which teams benefit from PIPs' engagement. For instance, typically, the PIP was identified through the team lead. Several PIPs and team leads shared how pre‐existing relationships supported greater PIP engagement as the team leads were able to leverage their existing rapport and identify, and prompt for, ways the PIP could meaningfully contribute. However, even in the absence of pre‐existing relationships, PIPs found it helpful to have their input explicitly requested. A few PIPs shared that, ideally, it would be helpful to receive advance notice that their input would be solicited in an upcoming meeting. For instance, one PIP shared, ‘If there was times where it was like, “Hey, [PIP name], this is what we're gonna be talking about. This is what we're gonna need your opinion on….I know that…they're gonna specifically be talking about this. And this is something that I can hyper, focus on and make sure that I have something to contribute to the conversation. So I'm not just sitting there on silent”.’ PIPs indicated this advance warning would help them recognize when they could contribute while also enabling time for preparation so that they could feel informed on a topic or gather their thoughts around an area. By having their perspective explicitly requested, it would also help them to feel valued. Several PIPs also indicated an appreciation for early and ongoing invitations for involvement in team discussions and decision‐making processes; such inclusion helped them understand the project aims, know where to focus their attention, and participate in opportunities to shape the team's work, rather than just reactively respond to the team's efforts.

Most PIPs expressed appreciation for being part of such an active, passionate, multidisciplinary team, but a few expressed some concerns with participating within the larger group context. One PIP indicated that smaller breakout groups sometimes helped make it clearer what they could contribute. Similarly, one team lead shared that in some cases, having smaller check‐ins where input is solicited may be more comfortable for the PIP, and a better use of the PIP's time. These check‐ins could provide an opportunity for questions and to get the PIPs input; one PIP expressed this, ‘Maybe even a check‐in – would be helpful. “Can I help explain what you just heard?” Or “how are you feeling about that?”’ A few PIPs suggested that one‐on‐one check‐ins may help them adjust to working in a different professional context and overcome ‘imposter syndrome’ when most of their non‐PIP teammates have graduate degrees. One PIP expressed this, ‘I'm not a doctor. I don't have a PhD… I don't even have my bachelor's yet…There are some things that are gonna go right over my head, and maybe a check in like… “Can I help explain any of what you just heard, or you know? Or how are you feeling about that?” Like that kind of thing.’ In some instances, PIPs questioned whether they were doing as much as they should be; they expressed that receiving more clearly articulated expectations at the start of the project regarding time, content, role, and resources, would help them to feel more confident that they are contributing as the role intends. Finally, a few PIPs suggested changes in the structure or labeling of the PIP role. One PIP suggested reframing the role as a consultant as that, ‘empowers individuals to embrace, giving feedback more…they have something to contribute as an equal…may help to feel they have equal weight of their voice to the conversation’.

Further, in the initial wave of Incubator teams, the PIP role was originally designed as a single person who brought the patient perspective to the team; on several subsequent teams this evolved into multiple PIPs. The inclusion of multiple PIPS limited any sense of tokenism, provided multiple perspectives, and created more continuity in the representation of lived experience perspective during team meetings when scheduling conflicts arose or when a PIP had to step away from the team. PIPs who were not the lone PIP on the team shared appreciating the additional contributions from other PIPs and indicated they felt there were still enough opportunities to contribute and feel valued.

*2b. The Impact of Virtual Meeting*

Most team meetings occurred via Zoom, and PIPs recognized both advantages and limitations ofvirtual connection. In terms of advantages, one PIP shared, ‘everyone being virtual made it easier tofeel just as much a part of the team as everyone else’. However, several PIPs expressed a desire for more in‐person opportunities to connect. One PIP, while acknowledging the benefit of virtual meetings removing attendance barriers, indicated that in‐person opportunities were more conducive to connection, relationship‐building, and increased comfort; these traits were described as potential facilitators for PIPs to share about their personal vulnerabilities and struggles among a group of unfamiliar individuals. One PIP shared, ‘virtual didn't detract from quality/connection, but in‐person would have been icing on the cake.’

*2c. Adjusting to Different Norms and Schedules*

Several PIPs expressed experiencing uncertainty in the beginning of the project regarding knowing what to do, or what their role should look like, and challenges adjusting to a new project and different professional setting. One PIP expressed this as, ‘a huge gap to catch up on, knowing how to relate and interconnect within a professional setting.’ Another PIP touched on this being a two‐way street, that not only did she have to adjust to team norms, but the team needed to adjust to her norms; for example, she described how her team needed to recognize the challenge that last‐minute cancellations created for people who do not have a 9–5 work schedule. Scheduling challenges were identified by several PIPs; one PIP suggested it may be worth exploring buying out time from their work organization to reduce work‐related conflicts, rather than utilizing the regular stipend approach. In a few instances, however, payment had to be waived or carefully constrained to avoid individuals losing any benefits that might be tied to income. Finally, a few PIPs touched on language challenges and differences in professional literacy; as one PIP indicated, ‘we don't speak the same language as the researchers do’ and another indicated, ‘I think I would have wanted a glossary, particularly of design‐thinking vocabulary.’ Several PIPs (and many team members in general) also had challenges accessing and navigating the Incubator's cloud‐based shared file storage platform which made accessing written information and storing project information a challenge. With help from team members, this was ultimately overcome.

In a few project teams, the PIP held a dual role, for example, they had lived experience but also played another role within the medical center. This duality had both strengths and challenges. As a strength, it provided more familiarity with the context of the work and may have reduced a sense of intimidation of participating in clinical‐academic teams. Further, PIPs who had a clinical role were able to participate in more diverse ways; whereas there were a few instances of PIPs who would have liked to contribute in more diverse ways but were limited by patient confidentiality and Health Insurance Portability and Accountability Act of 1996 (HIPAA) rules that prevented them from engaging directly with patients. As a challenge, however, sometimes the dual role created additional scheduling conflicts; additionally, when the PIP put on their workplace hat during meetings, it occasionally obscured their lived perspective and created uncertainty as to whether to respond as a healthcare service provider or as a patient. As a consequence, there was a request for, ‘maybe more clearly defining roles and expectations… just to know, like what's fully expected, and how they can be the most helpful.’

#### 3.2.3 Theme 3: Recommendations to Future PIPs

When asked to provide advice for future PIPs, the suggestions typically focused on the importance of being willing to participate and feeling empowered to share, recognizing the value that lived experience can bring to the team, and the general belief that ‘you get what you give’ and to ‘go all in.’ Several PIPs emphasized the need to fully engage and recognize their own value and the insights they can contribute, especially within ‘high‐powered’ teams consisting of doctors, faculty, and others with advanced formal education and familiarity with clinical and academic environments. As one PIP suggested, ‘Own what you do have to contribute. Be present, be assertive if you have something to say, work hard to feel like an equal member of the team. And don't do it if you are not going to fully commit.’ Another PIP stated, ‘Be sure to be involved as much as possible, ask questions, offer opinions, be a part of things, don't sit back because you are a patient and not a doctor…Doctors need help with [understanding] what the patient is experiencing.’ Others talked about their perspectives being accepted by their team and suggested, ‘Be open and tell them how you think, when you are thinking different things.’

Several PIPs also spoke about the emotional challenges of participation, noting that it can be “emotionally taxing” as it requires reflecting on their own, sometimes negative, prior experiences. They advised future PIPs, ‘Don't be afraid to take a break’ when needed, with one PIP expressing, ‘[Future PIPs] should be prepared for it to be hard sometimes to talk about things that impact them personally in front of people they don't know very well.’

## 4 Discussion

### 4.1 Recognizing and Maximizing Benefits for PIPs

The debriefing interviews highlighted the bidirectional value of lived experience engagement. These findings suggest that it is important to consider not just how research, quality improvement, and innovation projects can benefit from lived experience engagement, as typically emphasized in previous evaluations of patient engagement [1, 15], but also how the patient can benefit from participation. This includes, but also extends beyond, patients' gratitude for contributing to positive change that has been previously identified [16]. By recognizing and acknowledging the bidirectional opportunities and rewards that patient involvement can provide, individuals seeking patient input can be better equipped to identify potential patient partners and overcome well‐intended but exclusionary concerns about burdening patients who may already have health or social challenges. Further, teams integrating patient partners should continue to explore ways to maximize the mutual benefits of patient engagement beyond financial compensation alone and prevent exploitative dynamics. Examples include helping to navigate new professional settings, identifying how to apply newly gained skills in other contexts, and providing formal professional recognition of their contribution (i.e., letter detailing role, resume citation guidance). A prior review of patient‐compensation approaches in health research highlighted several nonfinancial forms of compensation, including coauthorship and informal acknowledgments on research outputs; however, effort to align the appropriate compensation approaches with what a given PIP values are likely needed [8].

To maximize the benefit of patient engagement—both for the patient and for the team—it is important to really get to know the PIP, including their strengths, interests, and positive and negative experiences, so that they can contribute fully and so that the team's work can be rewarding to them as well. In some instances, a pre‐existing relationship between the team lead and PIP jump‐starts this familiarity; in others, it remains especially important to develop personal connections and take opportunities to reach out and connect both within and outside of weekly team meetings on an ongoing basis, ideally, with at least occasional opportunities for in‐person team connection. One potential strategy to support getting to know the PIP and how they want to contribute is through a preference for involvement matrix, which provides a structure to help patients think about and express their preferred level of involvement throughout the stages of a project [17]. Similarly, checking in with PIPs at recurrent times during the project period, both informally, and perhaps through more structured formats (e.g., engagement surveys), will enable teams to adjust their approach and supports to optimize the benefits of PIP engagement [18]. Finally, teams should assess the long‐term impact of PIP engagement, including outcomes for PIPs (e.g., career advancement via project involvement) and for the innovation project.

### 4.2 Ameliorating Challenges by Highlighting and Clarifying Value

The interviews also revealed some of the challenges PIPs encountered through their project participation. Several PIPs spoke about adjusting to a different work setting and needing to recognize how and when their voice enhanced the conversation, with some initial sense of intimidation in joining teams that are largely comprised of individuals with graduate degrees. Team leads can validate PIPs' lived expertise and foster psychological safety to help with this adjustment by explicitly requesting input, and, when foreseeable, providing PIPs with advanced notice of the request for input so that they can reflect in advance of the meeting; prior research similarly highlights the value of clearly outlining expectations and meeting‐prework [19].

Recognizing the important role that language and labels can play, a few PIPs suggested that relabelling the role as a ‘lived expertise consultant’, rather than as a ‘patient innovation partner’, may help PIPs to recognize their own expertise, clarify the role, and inspire confidence to share their views. In addition, a focus on lived experience rather than being a patient may be more inclusive and inviting of individuals with diverse experiences and help to reduce potential stigma and the perceived power differentials associated with being a patient. However, such changes should be considered thoughtfully in partnership with PIPs to be sure that the sense of partnership is maintained and potential unintended adverse effects (e.g., imposter syndrome) are minimized. Prior work has similarly highlighted the importance of these terminology choices, recognizing the need to be flexible and understand the preferences of the individuals who share their personal expertise in this work [20]. Regardless, when framing the compensation provided to PIPs, it is important to acknowledge payment is not just a token appreciation for individuals' time, but also a recognition of their expertise and valuable contributions to the project. The importance of role clarity and clear expectations are also highlighted in other research as factors that enable patients to contribute in their desired fashion [21, 22].

### 4.3 Ameliorating Challenges Through Transparency and Support

Further, remaining cognisant of when acronyms or medical or research terminology are being used, and pausing to provide definitions and encourage questions, will enable PIPs to participate more fully [23]. Offering additional training on navigating research processes and ethical considerations may also be appreciated by many individuals new to the academic‐medicine work context. In addition, team leads should remain sensitive to the emotional toll and potential for re‐traumatization that participation may take on PIPs. To mitigate this risk, it is critical that teams create a safe and supportive environment, offering PIPs personalized support, providing linkages to mental health resources when appropriate, proactively building in flexibility to support PIPs to take time away if needed, and considering the inclusion of multiple PIPs when possible. Being cognizant of how best to support PIPs, reflecting on how the engagement is going, and acknowledging and addressing sources of discomfort will help to ensure the experience is positive for the PIPs—and for the full project team [24].

### 4.4 Looking to the Future

The debriefing interviews highlighted the potential for numerous implementation advantages, including enhanced acceptability, feasibility, fit, and reach of the innovation work [25]. The potential for PIPs to become ambassadors who can share personal accounts of the clinical/institutional commitment to the community and offer a peer's perspective on the value of new healthcare offerings among community members with shared experiences has the potential to reduce mistrust and barriers to accessing care and bridge the divide with the target population. While this is likely valuable across conditions, it is especially important for marginalized or stigmatized populations. Given these potential advantages, future work should explore PIP participation's impact on the implementation and dissemination of healthcare innovations. Further, given the inherent uncertainty involved in innovation work, where next steps are not prescribed, teams with PIP participants need to recognize that efforts to provide clarity, comfort, and assurance should be ongoing throughout the project; as goals and work evolve, PIPs' contributions will as well. Innovation work moves quickly; taking the time to slow down and form authentic relationships where diverse perspectives are valued, encouraged, and incorporated will increase the likelihood that true innovation and not just improvement occurs. This likely requires setting aside time to explicitly reflect and focus on the PIP relationship and opportunities for engagement and mutual benefit, working to develop a partnership where PIPs are meaningfully engaged throughout and not just consulted at specific points in the project [24, 26, 27]. Future work should continue to explore how best to support PIPs to be comfortable and fully contribute, while also supporting teams in how best to create an environment that is both receptive and encouraging of these contributions.

### 4.5 Limitations

This study had several limitations. The interviews that informed this project work were conducted immediately following the completion of each teams' project year. In the future, it may be helpful to share the questions in advance and offer a delayed post‐participation interview to enable PIPs the opportunity to reflect further on the experience and identify any personal benefits that may have been experienced following the project. Teams should also consider conducting additional interviews earlier in the project (e.g., at the mid‐point) to allow for responsive changes to support patient engagement. In addition, all interviews were conducted by Incubator staff, which could have made interviewees hesitant to express criticism. Interviews were also conducted to provide feedback to guide the Incubator's improvement; while PIP engagement was an explicit focus of this feedback, the inclusion of other topics may have limited the degree to which responses were probed and consequently, the depth in which some respondents replied. Further, it is important to acknowledge that these insights are based on a small sample that was predominantly female and Non‐Hispanic White; further research is needed to explore the experiences of individuals with more diverse backgrounds and healthcare challenges. Finally, much of this work was conducted in the height of the COVID‐19 pandemic, and perspectives on the pros and cons of virtual engagement may have shifted over time [28].

## 5 Conclusion

Despite the limitations, this work offers valuable insights into the benefits of patient engagement in healthcare innovation teams and offers important considerations for how to structure and support PIP roles. When the relationship between the PIP and the team is fostered and supported, there are numerous and far‐reaching benefits. While there is likely not a one‐size‐fits‐all approach, the hope is that this manuscript will help future researchers and individuals engaged in improvement efforts think thoughtfully and make intentional decisions to incorporate lived experience perspectives into their work—and recognize and facilitate the opportunities for mutual benefit from such engagement.

## Author Contributions

**Shoshana H. Bardach:** conceptualization, writing – review and editing, writing – original draft, formal analysis, methodology, investigation, project administration, data curation. **Amanda Perry:** conceptualization, data curation, formal analysis, writing – original draft, methodology, investigation, project administration, writing – review and editing. **Terry Sturke:** writing – review and editing, formal analysis. **Randy Stevens:** writing – review and editing, formal analysis. **Milan F. Satcher:** formal analysis, writing – original draft, writing – review and editing.

## Ethics Statement

This work was reviewed by the Dartmouth‐Hitchcock IRB and was determined to be nonhuman subjects research and considered part of a quality improvement initiative.

## Conflicts of Interest

The authors declare no conflicts of interest.

## Data Availability

Due to small numbers of individuals interviewed and the ability to re‐identify based on transcript data, supporting data are not available.
